# Adenoviral CD40 Ligand Immunotherapy in 32 Canine Malignant Melanomas–Long-Term Follow Up

**DOI:** 10.3389/fvets.2021.695222

**Published:** 2021-07-23

**Authors:** Sara Saellstrom, Arian Sadeghi, Emma Eriksson, Thomas Segall, Maria Dimopoulou, Olle Korsgren, Angelica SI. Loskog, Thomas H. Tötterman, Akseli Hemminki, Henrik Ronnberg

**Affiliations:** ^1^University Animal Hospital, Swedish University of Agricultural Sciences (SLU), Uppsala, Sweden; ^2^Department of Immunology, Genetics and Pathology, Uppsala University, Uppsala, Sweden; ^3^National Veterinary Institute, Department of Pathology and Wildlife Diseases, Uppsala, Sweden; ^4^Cancer Gene Therapy Group, Translational Immunology Research Program, University of Helsinki, Helsinki, Finland; ^5^Comprehensive Cancer Center, Helsinki University Hospital, Helsinki, Finland; ^6^Center of Clinical Comparative Oncology (C_3_O), Department of Clinical Sciences, Faculty of Veterinary Medicine and Animal Science, Swedish University of Agricultural Sciences (SLU), Uppsala, Sweden

**Keywords:** immuno oncology, adenoviral vectors, translational medicine, canine malignant melanoma, clinical trials, AdCD40L

## Abstract

Malignant melanoma is a serious disease in both humans and dogs, and the high metastatic potential results in poor prognosis for many patients. Its similarities with human melanoma make spontaneous canine melanoma an excellent model for comparative studies of novel therapies and tumor biology. Gene therapy using adenoviruses encoding the immunostimulatory gene CD40L (AdCD40L) has shown promise in initial clinical trials enrolling human patients with various malignancies including melanoma. We report a study of local AdCD40L treatment in 32 cases of canine melanoma (23 oral, 5 cutaneous, 3 ungual and 1 conjunctival). Eight patients were World Health Organization (WHO) stage I, 9 were stage II, 12 stage III, and 3 stage IV. One to six intratumoral injections of AdCD40L were given every seven days, combined with cytoreductive surgery in 20 cases and only immunotherapy in 12 cases. Tumor tissue was infiltrated with T and B lymphocytes after treatment, suggesting immune stimulation. The best overall response based on result of immunotherapy included 7 complete responses, 5 partial responses, 5 stable and 2 progressive disease statuses according to the World Health Organization response criteria. Median survival was 285 days (range 20–3435 d). Our results suggest that local AdCD40L therapy is safe and could have beneficial effects in dogs, supporting further treatment development. Clinical translation to human patients is ongoing.

## Introduction

Canine melanoma is a cancer presenting in many different forms, each with different prognosis, just like its human counterpart. Prognosis is dependent on anatomical location, stage at clinical presentation and histologic grade. Oral and digital (ungual) malignant melanomas carry the worst prognosis with a metastatic rate ranging from 40 to 100% at initial diagnosis and a median overall survival (OS) after radical surgery of less than five months (1–3). Cell proliferation is reported to predict survival and a mitotic index >2 is considered of prognostic significance (3). In contrast to humans, where most melanomas in the skin are classified as a skin cancer (4), only about 20% of dermal melanomas in dogs are malignant, but the high-grade types still result in a poor prognosis. Ocular melanomas are in general benign, with a few important exceptions where severe aggressive behavior can be seen, and also here mitotic index seems the most reliable prognostic indicator (5, 6). Histological grade, in particular nuclear atypia and proliferation index using Ki-67, has been shown to correlate to prognosis (7, 8). Median survival times for dogs with oral melanoma treated with surgery are approximately 17–18, 5–6, and 3 months with stage I, II, and III disease, respectively, clearly indicating that there is an unmet need for developing adjuvant treatment modalities in high-stage disease. Aggressive facial and digital surgery in dogs can severely influence normal function and quality of life post-operatively. A multimodal therapy approach can therefore contribute to decreased surgical intensity, while still extending overall survival with fewer adverse events, for improved animal welfare. Spontaneous models of tumors in pet dogs have been receiving increased attention as a comparative asset for many cancers in humans, including malignant melanoma (9–11).

Immune evasion is one hallmark of cancer and is identified in both human and canine malignant melanomas (12–14). Several mechanisms are involved in this process, including T regulatory (Treg) cell expansion, myeloid derived suppressor cells, (MDSC), M2 tumor-promoting macrophage switch and immunosuppressive cancer-derived exosomes (15, 16). Immunoediting is a stepwise process and can be evolved over several years (17). In general, the cancer tries to induce and maintain a chronic inflammatory state and suppress signals and pathways, creating an acute inflammatory environment (18, 19). The role of dendritic cells is crucial for genesis and maintenance of the tumor (20). CD40 is a co-stimulatory molecule belonging to the tumor necrosis factor superfamily and is essential in activation of dendritic cells (21). In many tumors this expression is suppressed and the dendritic cells are inactivated (22). Hence, reactivation of dendritic cells with CD40 upregulation is an attractive strategy in immunotherapy of different tumor models *in vitro*, in animal models and in human clinical trials. Adenoviral cancer therapy has long been recognized as a promising approach and has developed from monotherapy gene transfer into a combinational approach. Here oncolytic genetically modified adenoviral systems generate neoantigen exposure to the tumor-associated immune system. The transgene product simultaneously activates the immune system suppressed by the tumor. Adenoviral cancer therapy is generally seen as an attractive system because of low toxicity with a convenient and reliable system of gene editing and gene expression, and was the first viral vector developed for gene therapy, being approved for clinical trials in 1990 (23). Even if only a small part of the tumor becomes infected, the cascade developed in the tumor can still be sufficient to induce a tumor response as well as creating tumor-specific immune cells that can move away from the treated tumor and seek and destroy cancer cells in other locations, and hence an abscopal effect is generated.

Malignant melanoma in humans (HMM) often occurs in the skin, but it originates from the neural crest; hence malignant melanoma also can occur on mucous membranes and in the gastro-intestinal canal, as well as the CNS, here primarily as metastases. Local low-stage disease has an excellent prognosis, whilst the 10-year survival in stage IV melanoma is only 10%. Canine malignant melanomas are in many aspects suggested as a good comparative model to HMM, particularly the rare non-UV-induced human melanomas, especially mucosal melanomas (10). *In vitro* stimulation in malignant melanomas as well as intratumoral activation of CD40 in melanoma in mouse models have shown promising results (24, 25). Two earlier studies of replication-deficient AdCD40L intratumoral treatment of spontaneous canine malignant melanoma have been reported (26, 27). Hence, the rationale is obvious for perusing human malignant melanoma in a clinical setting (28, 29).

The aim of this current study was to include a larger number of dogs than previously reported, and with a life-long follow-up, to present survival data and finally identify long-term effects of episomal CD40L gene therapy.

## Materials and Methods

### Study Design

This is a long-term follow-up study of a pilot study using AdCD40L immunotherapy. In addition to the 19 previously reported dogs (26), 13 additional dogs were added and all dogs were followed up until death. This data was collected by contacting the dogs' owners, as well as retrieving individual patient information available in the electronical medical record system (Trofast, Sanimalis, Sweden) at the University Animal Hospital UDS, SLU. Owners were asked for the date on which their dog passed away/was euthanized, the reason for ultimate euthanasia, and if recurrence of melanoma was known to have occurred.

Between May 2005 and May 2013, 32 client-owned dogs with spontaneously occurring malignant melanoma participated in a study of local intratumoral AdCD40L immunotherapy. AdCD40L therapy and sample collection were approved by the Swedish Animal Ethical Committee and the Swedish Animal Welfare Agency (C228/6). A written consent form from the dog owners was obtained. The therapy was performed within the University Animal Hospital (UDS), at the Swedish University of Agricultural Sciences (SLU).

Dogs were treated with weekly intratumoral and/or metastatic lymph node injections (*n* = 1–6) of a human adenovirus serotype 5 carrying a human CD40 ligand administered under subcutaneous sedation with medetomidine/butorphanol (0.01 mg/0.1 mg/kg), with local lidocaine (100 mg/ml) anesthetic spray for oral treatments. All sedated animals were reverted with intramuscular injection of atipamezole (0.05 mg/kg) post AdCD40L injection.

At each treatment, blood samples were collected; CBC, standard clinical chemistry profile, and immunoglobulin gel electrophoresis were performed. The AdCD40L vector was prepared by diluting 37 μl of stem solution (viral titer 7 × 10^10^ Pfu/ml) to a volume of 1 ml (2.6 × 10^9^ Pfu) with sterile isotonic sodium chloride. This GMP-produced AdCD40L was constructed at Baylor College of Medicine, Huston, TX, USA and full description of the virus is described in (30). Between 1 ml and 3 ml AdCD40L were injected each time using a syringe and a 25-gauge needle.

Being a toxicity study on AdCD40L administration in dogs with high stage/high grade malignant melanomas, initial dogs started on low doses and few injections, carefully increasing the dose (volume and titer) to monitor any toxicity. As the toxicity did not differ between 0.5 and 3 ml, it was decided to standardize the dosing regimen to 1.0 ml x 3. Exceptions were made where owners insisted to have additional treatments as the vaccine had a positive effect on tumor burden and quality of life. After an initial dose-escalation phase, the suggested dose (1 ml−2.6 × 10^9^ Pfu), was considered optimized, as a larger volume could be difficult to administered in smaller lesions. The protocol also is analog to human trials, where the final selected dose is used, regardless on tumor size or weight of the patient. In some trials, the full dose is given in one selected lesion and in other protocols the dose can be diluted and divided into several lesions. In our study, a split dose regime was used. Hence, the dose volume was adjusted with dilution buffer to render the same injection volume per tumor lesion for split dose treatments as for full dose treatment. Detailed information on oncolytic viral dose calculation in human malignant melanoma is described in (31). Although, the study is using a conditionally replicative oncolytic herpes virus, it is still instructive and pivotal in explaining principles in dose calculation regardless of tumor or patient size and is the first FDA approved oncolytic virus therapy for human refractory melanoma (T-VEC, or Imlygic®).

Tumor tissues sampled pre- and post-treatment were investigated for immune cell infiltration in 20 of the dogs. Histological staining was performed according to the method described previously (27). Briefly, tumor specimens were fixed in a buffered 10% paraformaldehyde solution and paraffin embedded. Tissue sections were prepared from the paraffin-embedded tissues for routine hematoxylin-eosin staining and immunohistochemistry and subjected to staining procedures with either monoclonal mouse anti-human CD3 (clone F7.2.38, DakoCytomation, Glostrup, Denmark) or monoclonal mouse anti-human CD79 α (clone HM57, DakoCytomation). The complex expressions were visualized by using the DakoCytomation EnVision+R system HRP anti-mouse (K 4001) with diaminobenzidine as substrate. Nuclear counterstaining was performed with Mayer's hematoxylin. Investigations were done according to histologic remission and immune cell infiltration. Briefly, histologic remission means less viable tumor cells present after treatment and histologic complete remission—that is, no tumor cells present post treatment (or post mortem). Immune cell infiltration was graded 0–3, with 3 being the highest grade of infiltration. Macroscopic tumor immune reaction was clinical observation of swelling at the injection site, ulceration and regional lymphadenopathy occurring days post immunotherapy.

### Clinical Assessment of Response

**Figure 1:**
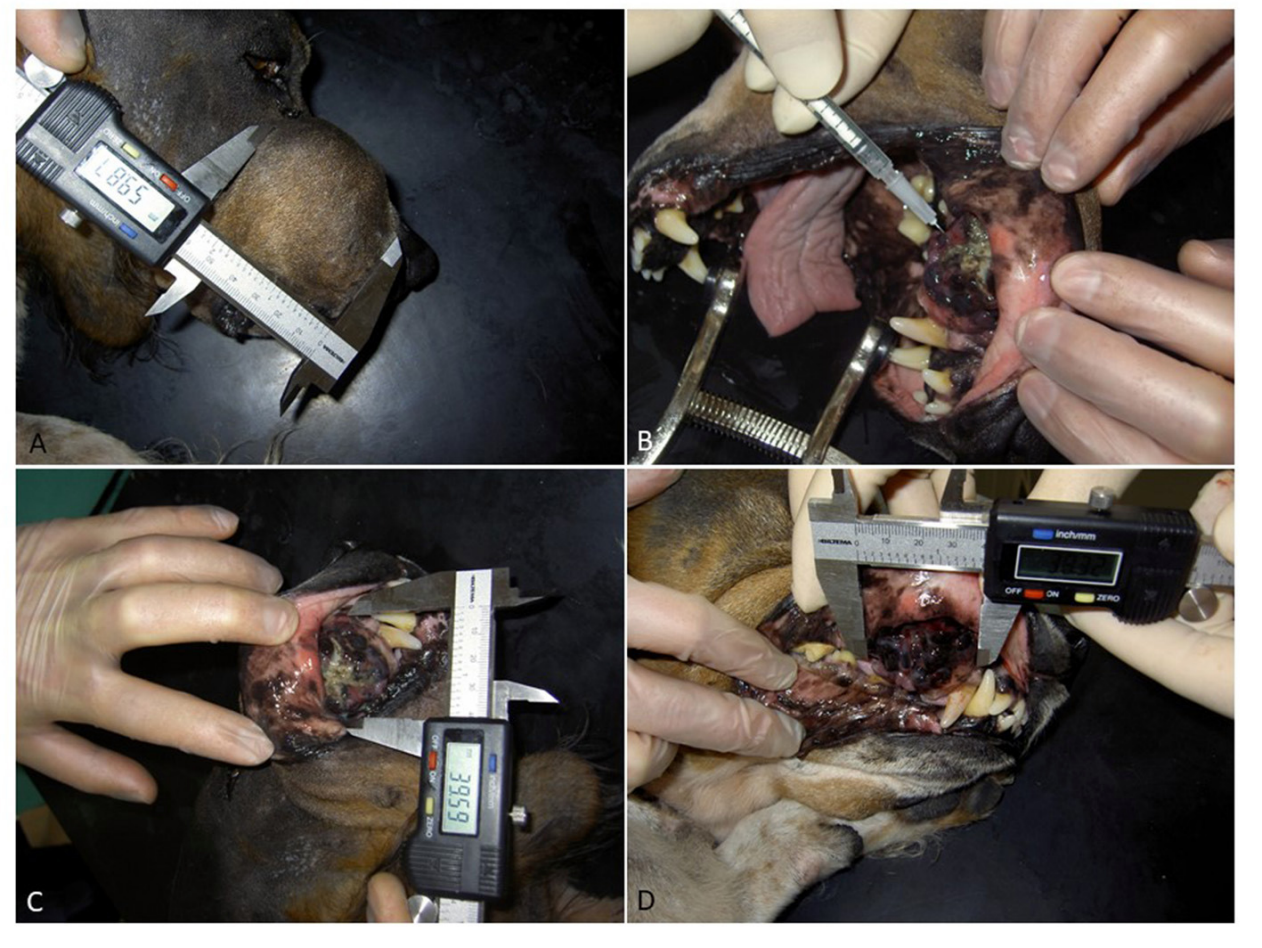
A dog with oral malignant melanoma was presented with bone involvement and lymph node metastases and was considered refractory to surgery. **(A,B)** Tumor size measurements were made with a digital caliper and documented with photography with dogs under sedation. Picture **(C)** shows the first injection of AdCD40L. Picture **(D)** shows the second injection of AdCD40L, 7 days after first injection. Please note the rapid organization of the tumor and significantly less necrotic tissue. The dog started eating as soon as 2 days post first injection, clearly showing an improved quality of life.

Response to therapy was categorized in accordance with WHO criteria, where the products of bidimensional lesion measurements are summed and the change is calculated from baseline while on therapy in order to quantify response (32). More specific CR is specified as complete regression of measurable soft tissue disease, PR (partial regression, >50% but <100% regression in one dimension of measurable soft tissue disease), SD (stable disease, ≤50% regression of soft tissue disease or ≤25% progression), and PD (progressive disease, >25% increase in measurable disease or appearance of new lesions). The minimal time interval required between two measurements for determination of stable disease response was 6 weeks. Best overall response was defined as the best response recorded from the start of the treatment until disease progression or recurrence. Macroscopic tumor lesions were measured by digital caliper and documented with photography (Figure 1). In addition, dogs were followed with diagnostic imaging (computer tomography, radiography and/or ultrasound), using the best suitable modality for each case. Treatment adverse events were graded according to the VCOG-CTCAE, version 1.1 (33).

Ultrasound was used very infrequent to follow tumor response (most commonly to follow non-target lesions or for staging purposes) and used only if any other modality was considered inappropriate. If used, the guidelines described in (34) was used, and more specific, the same machine and operator performed all examinations for the individual patient. Dogs were rechecked for as long as clinically necessary to determine survival for each dog. Overall survival was defined as the time from first treatment with AdCD40L until subsequent death. All dogs were followed up until death.

### Statistical Analyses

Survival-time distributions were estimated using the Kaplan–Meier method. Statistical analyses were carried out using JMP Pro 15.2.1 (SAS Institute Inc., Cary, NC) statistical discovery software. Univariate survival analysis was carried out for each parameter. Parameters determined to be of statistical significance in the univariate analysis according to log-rank testing were used to stratify Cox proportional hazard models. Parameters included in this study were weight, sex, breed, WHO stage, melanotic vs. amelanotic primary tumor, primary tumor location, presence of local or/and distant metastases, number of AdCD40L treatments, adverse effects after treatment, response, surgery after treatment initiation, histologic tumor type and grade. A parameter with a *P*-value <0.05 was considered significant. Adjusting for multiple testing was performed by Bonferroni correction (35). The Bonferroni correction compensates for that increase by testing each individual hypothesis at a significance level of α/m, where α is the desired overall α level (0.05) and m is the number of hypotheses.

Kaplan–Meier survival graphs were calculated for stage, tumor grade, histological type, weight and response, as well as for patients who underwent surgery in combination with immunotherapy vs. patients who did not.

For the purpose of this study, tumors were histologically described as being melanotic if any part of the histological sample was deemed to contain pigment. Multiple tumors which were histologically described as “mainly amelanotic” have therefore been classified as melanotic for the purposes of statistical analysis. For the purpose of WHO staging, ungual and conjunctival tumors were classified according to the protocol for dermal or epidermal tumors, because of the lack of a universal staging system for tumors of these locations. Patients were also grouped according to weight and stage for statistical analyses. Weight was divided into two groups: <20 kg (*n* = 17) and >20 kg (*n* = 15). Similarly, patients were subdivided into three age groups: <9 years (*n* = 11), 9–11 years (10), and >11 years (*n* = 11).

When analyzing histologic cell type, groups were dived into epithelioid, spindle shape, mixed and balloon cell (clear cell) melanoma. Grade was divided into low and high, with mitotic index above 2 as the determinant.

## Results

### Patient Population

The patient population consisted of 32 dogs with spontaneously occurring malignant melanoma: 22 oral, 5 cutaneous, 3 sub ungual, 1 nasal and 1 conjunctival (Table 1). Patients were staged according to the WHO staging system (36). Of the patients included, 8 were stage I, 9 stage II, 12 stage III and 3 were stage IV. Median age at the start of therapy was 120 months (48–168 months). Median weight was 32.2 kg (5–49 kg). Thirteen of the dogs were intact female, 3 were spayed females, 13 were intact males and 3 were neutered males. Twenty of the 32 dogs underwent surgery, 7 before immunotherapy was initiated and 13 after. Three dogs underwent surgery both prior to and after therapy initiation. The vast majority of surgeries performed were cytoreductive and not radical with curative intent. Two dogs underwent a second surgery post AdCD40L immunotherapy after initial unclean margins. At second surgery no remaining tumor cells could be detected and the margins were clean, thus we classified only these two surgeries as curative-intent. One dog had undergone chemotherapy as part of previous treatment, but tumor control had not been achieved. The number of AdCD40L treatments ranged from 1 to 6 with a mean of 3; 15 dogs got 3 treatments and an injection volume between 0.5 and 3 ml depending on tumor volume.

**Table 1:** Study demographics.

**Breed**	**Age (Y)**	**Weight (Kg)**	**Gender**	**Stage**	**Grade**	**Histologic type**	Treatment^*^	**BOR**	**Time Sx**	**Surgery**	**Survival (Days)**
Golden retriever	8	32	F	III	H	Spindle, amelanotic	1.5 ml X2	NA	9	Y	401
American cocker spaniel	11	12.5	M	I	L	Epithelioid	1,5–3 mlX5	NA	−30, 40	Y	1,141
Siberian husky	11	26.9	F	I	H	Mixed	1 mlX6	CR	85	Y	645
Dachshund	4	5.2	F	II	L	Epithelioid	1 mlX5	NA	−35, 37	Y	3,435
Irish setter	9	37.8	M	II	L	Epithelioid	1 mlX5	CR	81	Y	500
Golden retriever	12	32.8	F	III	H	Balloon cell	2 mlX4	NA	16	Y	130
Mixed	13	32.4	FS	III	H	Mixed, amelanotic	2 mlX3	NA	28	Y	180
Golden retriever	7	31	F	III	H	Epithelioid, anaplastic	2 mlX3	NA	23	Y	279
Irish setter	12	24	F	III	H	Epithelioid	2 mlX3	NA	25	Y	78
Mixed	11	36.5	F	I	L	Spindle	1 mlX3	CR	−30	Y	1,083
Flat coated retriever	4	37.5	M	I	L	Epithelioid	1 mlX3	NA	15	Y	2,057
Rottweiler	5	49	M	I	L	Mixed	1 mlX3	NA	−15	Y	495
Mixed	5	35	FS	I	L	Epithelioid (cytology)	1 mlX1	NA	15	Y	1,340
Golden retriever	12	33	M	II	H	Mixed	1 mlX3	CR	55	Y	225
Rottweiler	8	34.5	F	I	L	Mixed	1 mlX3	NA	35	Y	1,818
Golden retriever	14	31	M	II	L	Epithelioid	1 mlx3	CR	−30	Y	356
Labrador retriever	12	40	MC	II	L	Spindle	1 mlx6	CR	−46	Y	336
Giant poodle	14	13	FS	II	H	Epithelioid	1 mlx3	CR	248	Y	260
Risenschnauzer	7	47	F	III	H	Epithelioid	1 mlx2	NA	−7, 400	Y	500
Mixed	12	5	MC	II	H	Epithelioid	1 mlx3	NA	35	Y	308
Jack russel	9	7.8	M	I	H	Spindle	1.5 ml X6	PR		N	290
Basset artesién	14	15.4	M	IV	H	Mixed	0.5–2 ml X3	PR		N	60
Golden retriever	9	33.8	M	III	H	Epithelioid	2–3 ml X3	PD		N	100
Border terrier	12	9.9	M	III	L	Epithelioid	1 ml X1	SD		N	160
Flat coated retriever	8	39.6	M	III	H	Epithelioid	2 ml X5	SD		N	131
Swedish lapphound	11	23	M	III	H	Mixed	2 mlX3	PR		N	48
Danish-Swedish farmdog	9	9.2	M	III	H	Mixed	1 mlX3	SD		N	128
Risenschnauzer	8	38	F	IV	H	Mixed	2 mlX2	PD		N	20
German shepherd	14	40	MC	IV	H	Mixed	2 ml X2	PR		N	55
Pumi	13	11.2	FS	III	H	Mixed, amelanotic	2 mlX2	SD		N	41
Rottweiler	9	34	F	II	H	Mixed	1 mlX4	SD		N	42
Risenschnauzer	6	22	F	II	L	Epithelioid	1 mlx6	PR		N	1,211

*BOR, Best overall response; CR, Complete response; PR, Partial response; SD, Stable disease; PD, Progressive disease; NA, Not Applicable. Time of surgery (Sx) in days related to AdCD40L treatment. Some Sx were performed prior to immunotherapy. Two dogs achieved clean margins post 2nd Sx post AdCD40L treatment*.

* *Virus concentration was 2.6 – 10^9^ Pfu/ml*.

### Clinical Response to AdCD40L Treatment

**Figure 2:**
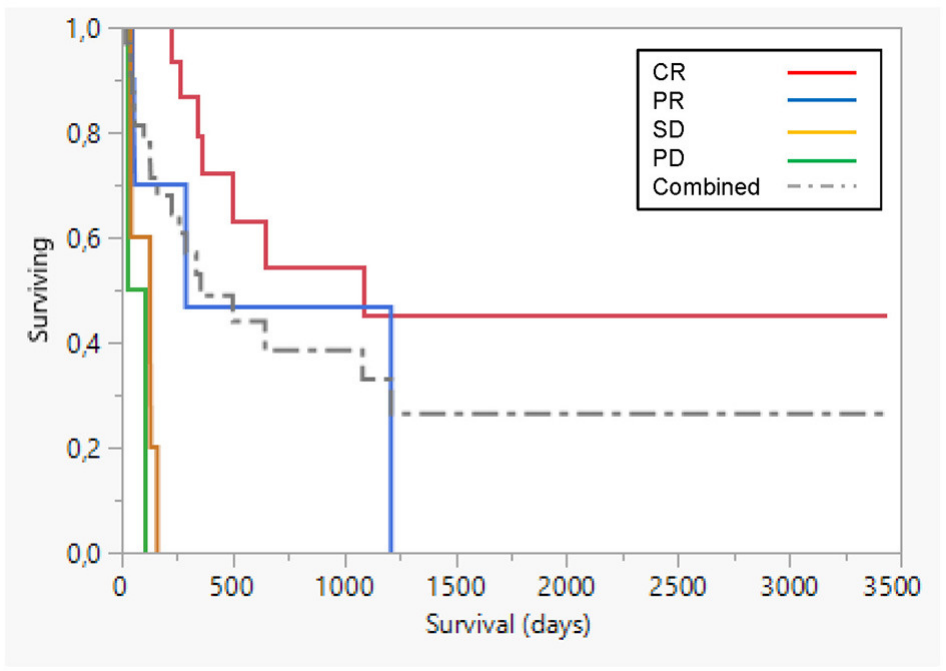
Survival plot comparing prognosis for different responses. Best overall response according to WHO response criteria, based on macroscopic change due to AdCD40L therapy was 7 complete response (CR; red line), 5 partial response (PR; blue line), 5 stable disease (SD; yellow line), and 2 progressive disease (PD; green line). Dogs achieving a complete or partial response had a significantly longer median survival (*p* < 0.0001). Dotted line combined group.

Best overall response according to WHO response criteria, based only on macroscopic change post immunotherapy was 7 complete response (CR), 5 partial response (PR), 5 stable disease (SD), and 2 progressive disease (PD) (Table 1). In all cases, a clinically evident inflammation occurred in the tumor after the course of AdCD40L injections, with initial edema often followed by increased firmness and signs of organization. Dogs achieving a complete or partial response had a significantly longer median survival (*p* < 0.0001) (Figure 2). Details related to survival compared with different responses to therapy are presented in Table 2.

**Table 2:** Details related to survival compared with different responses to therapy in 19 dogs with macroscopic response to AdCD40L therapy.

**Response**	**Number**	**Mean (D)**	**Std error**	**Median (D)**	**Lower 95%**	**Upper 95%**	**p**
CR	7	772^*^	104.6	1,083	336	·	<0.0001
PD	2	60	40	60	20	100	
PR	5	649^*^	239.7	290^*^	48	1,211	<0.0001
SD	5	100.2	24.7	128	41	160	
Combined	19	601	97	356	160	1,211	

*Dogs achieving CR and PR had a significant longer survival compared to the rest of the groups. CR, complete response; PR, partial response; SD, stable disease; PD, progressive disease*;

* *significant p < 0.008, Bonferroni correction for multiple comparisons*.

**Figure 3:**
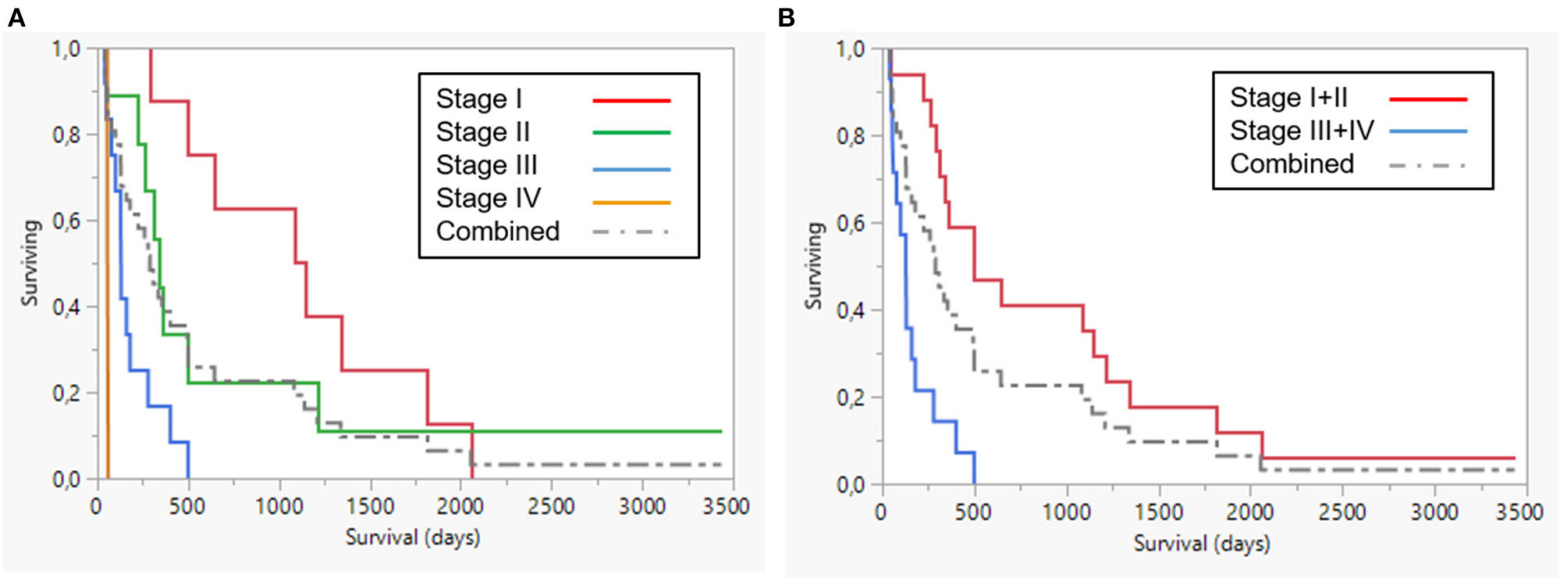
Survival plot **(A)** all stages **(B)** Stages I+II (red) and III+IV (blue). Dotted line combined group.

Overall median and mean survival times were 285 and 558 days, respectively. Median and mean survival for dogs in stages III–IV were 128 and 154 days (Figure 3). Median and mean values for all stages are shown in Table 3. Stage was significant, both as separate stages (*p* < 0.0001) with Bonferroni correction for multiple comparisons (*p* < 0.008), where stage III was prognostic, as well as where stage was grouped into low (I+II) and high (III+IV) respectively.

**Table 3:** Comparison of survival between different stages.

**Stage**	**N**	**Mean**	**Std error**	**Median (D)**	**Lower 95%**	**Upper 95%**	***p***
I	8	1108.6	220.4	1,112	290	1,818	
II	9	741.4	354.0	336	42	1,211	
III	12	181.3	41.1	131^¤^	48	279	0.0001
IV	3	45	12.6	55	20	60	
I+II	17	914.2	212.8	500	290	1,211	
III+IV	15	154.1	35.7	128^*^	48	160	0.0001
Combined	32	558	131.6	285	130	495	

*Stages also merged in low (I+II) and high (III+IV) to equal group size. Survival was significantly lower in stage III compared to all other groups. When only comparing low (I+II) and high (III+IV) stage, it was a significantly worsened prognosis in high stage*.

¤ *Significant, p < 0.008, Bonferroni correction due to multiple comparisons*.

* *Significant, p < 0.05*.

**Figure 4:**
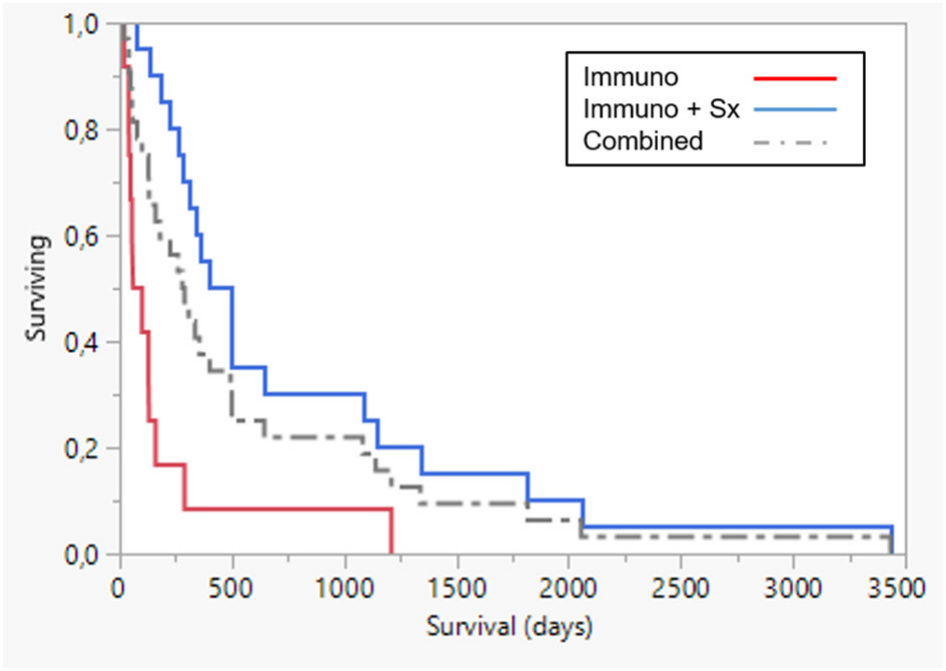
Survival plot comparing AdCD40L immunotherapy only (n = 12, red line), median 80 days and combined with surgery (Sx) (n = 20, blue), median 448 days. Survival was significantly longer in dogs that also underwent surgery combined with immunotherapy p < 0.0001. Dotted line combined group.

Median and mean survival times for patients who underwent surgery were 448 days (78–3,435) and 778 days respectively. For patients who did not undergo surgery combined with immunotherapy, median and mean survival times were 80 days (41–1,211) and 191 days respectively. Survival was significantly increased in dogs that also underwent surgery (*p* < 0.0001) (Figure 4).

Parameters significant in univariate survival analysis were breed, age, presence of local/distant metastases, histologic grade, tumor stage, tumor type, and surgery after therapy initiation. These parameters were then used to stratify a Cox proportional hazard model and significance remained for breed (Golden Retriever) (*p* < 0.00001), stage (0.00006), age (dogs below 9y lived longer) (*p* = 0.0119) and surgery together with immunotherapy (0.0001). All details regarding survival with or without combined surgery and AdCD40L treatment are shown in Table 4.

**Table 4:** Comparison of survival between dogs only treated with immunotherapy and dogs also undergoing surgery.

**Group**	**N**	**Mean (D)**	**Std error**	**Median (D)**	**Lower 95%**	**Upper 95%**	**p**
AdCD40L only	12	191	95.2	80	41	160	
Surgery plus AdCD40L	20	778	187.8	448^*^	260	1,083	<0.0001
Combined	32	558	131.6	285	130	495	

*Survival was significantly longer in dogs that also underwent surgery combined with immunotherapy (p < 0.0001)*.

* *Significant, p < 0.05*.

### Clinical Immunology

Immunological responses, based on serum cytokine analysis (TNF-α, IL-8, and the T-cell response suppressor IL-10) have been reported before for 19 treated dogs, including neutralizing antibodies in 100% post the third immunotherapy in six tested dogs (26, 37).

In one case where a metastatic prescapular lymph node was injected, distant immunoreaction was identified in a previously undetected metastatic lesion in the brain. This dog developed seizures three days after each injection with AdCD40L vector and was euthanized one week post second injection. The CNS metastatic lesion was examined post mortem and compared to adjacent normal brain tissue; the metastatic lesion was infiltrated with T-lymphocytes. The histologic findings, together with the clinical appearance of seizures occurring three days post injection, suggest abscopal effect of the AdCD40L injection.

### Safety and Toxicity

All adverse events were recorded. Adverse reactions (considered related to treatment) were seen in twenty out of thirty-two dogs. Mild transient fever developed in seven, and five had mild inappetence/anorexia. Twenty dogs had swelling at the injection site, and this was most prominent in dogs that received AdCD40L therapy in a metastatic lymph node. Five dogs had a mild and transient increase in liver enzymes after treatment; one had a grade 2 increase in both alanine amino transferase (ALT) and alkaline phosphatase, and another had a grade 2 rise in ALT (graded according to the Veterinary Cooperative Oncology Group Common Terminology Criteria for Adverse Events [VCOG-CTCAE] following chemotherapy or biological antineoplastic therapy in dogs and cats v1.1) (33). No other adverse effects were noticed. The adverse reactions after AdCD40L injection seemed to decline after one or two treatments, which corresponded to an increase in adenovirus-specific neutralizing antibodies in serum post therapy.

Due to the low toxicity and that no increase in toxicity could be registered even when the virus titer increased up to 3 ml (2.6 × 10^9^ Pfu/ml), and that 100% of the dogs developed neutralizing antibodies after three injections, it was decided to use a regimen of 3 times 1 ml injection, irrespective of tumor size or number of lesions in the latter half of the trial. Exceptions were made, where owners requested additional injections where a clear tumor response was achieved.

### Histopathology

Histological evaluation was performed in 31 dogs (one case only diagnosed with cytology), showing 15 epithelioid melanomas, 4 spindle cell melanomas, 12 mixed type melanomas and 1 balloon cell melanoma (Table 1). The histological diagnosis of melanoma was based on the histological classification of tumors in domestic animals according to cytological appearance, number of mitoses, pleomorphism, and presence of anaplastic and poorly differentiated cells (38). However, histological evaluation alone is not always a reliable prognostic indicator; rather the combination of oral location and morphology indicating malignancy support a worse outcome of melanoma in dogs. In the case where diagnosis was based on fine needle aspiration biopsy, a board-certified veterinary clinical pathologist analyzed the cytology and the tissue specimen was reported to contain a high frequency of mitoses and a round cell population of markedly pigmented cells. Thus, a tentative definition of epithelioid, high-grade malignant melanoma was made.

**Figure 5:**
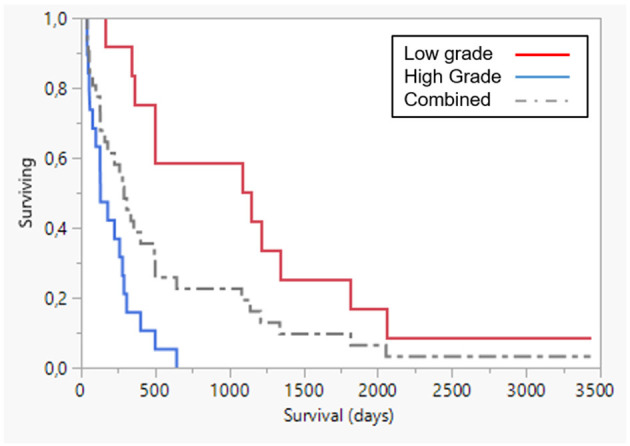
Survival plot: low grade (red line, *n* = 12) vs. high grade (blue line, *n* = 20) (*p* < 0.0001). Dotted line combined group. Survival was significantly lower in high grade disease.

**Figure 6:**
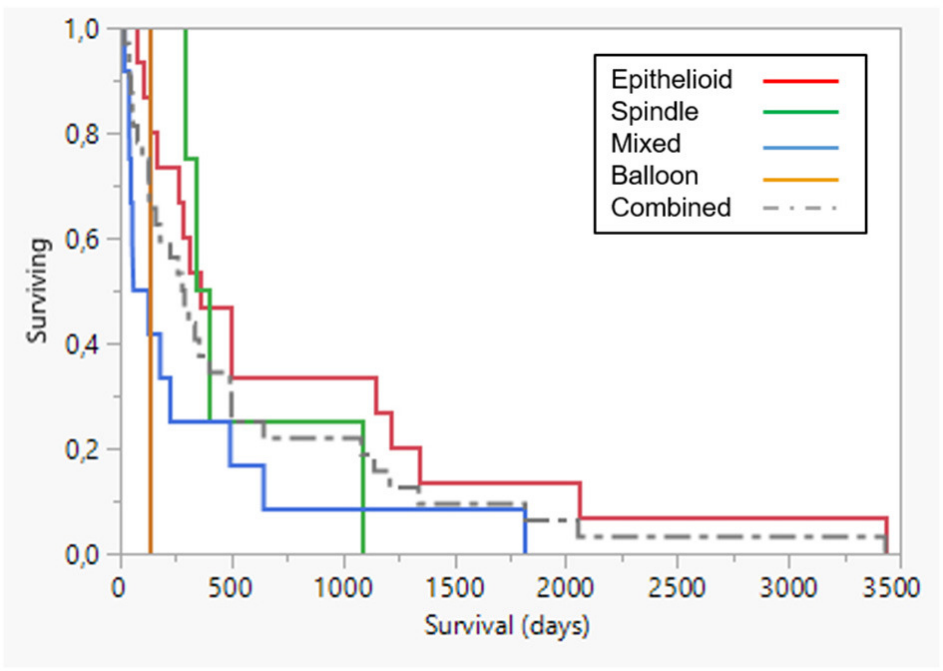
Survival plot comparing prognosis in different histological groups. Epithelioid (*n* = 15, red line), spindle type (*n* = 4, green line), mixed type (*n* = 12, blue line) and one balloon cell (clear cell) melanoma. Dotted line combined group. Mixed cell type had a *p*-value of *p* < 0.035, which was not considered significant when addressing the Bonferroni correction for multiple comparisons (*p* < 0.008).

Several publications report proliferation as a consistent prognostic marker of malignant melanoma in dogs. Hence, a grading into low and high grade was made with mitotic index (MI) of >2 per 10 high power field (HPF) as the determinant. With this subdivision, 12 tumors were low grade and 20 were high grade and this was significant (*p* < 0.0001) (Figure 5). For full details of survival data in different histology grades, see Table 5. Immune activation by AdCD40L therapy was seen in several of the patients. An increase in the infiltration of immune cells was seen in 8 of 11 dogs. Histological grading described in (26) was based on lymphocyte counts of random representative areas in the tumors, and defined as: none = 0, 1–10 lymphocytes per high power field = 1, 11–30 lymphocytes per high power field = 2, and >30 lymphocytes per high power field = 3. Tumor regression was seen histologically in 25 of 32 cases. There was an initial significant relationship between histological tumor type and overall survival, with the mixed type showing a worse outcome (*p* < 0.035), although with Bonferroni correction for multiple comparison (*p* < 0.008) this significance was lost (Figure 6). Details of the outcome depending on histologic type are presented in Table 6. No statistical significance was seen in the relation between the increase of immune cell infiltration after treatment and overall survival. A comparison was made on survival depending on melanotic (*n* = 25) or amelanotic (*n* = 7). No statistical difference on survival between the groups were seen (*p* < 0.11).

**Table 5:** Comparison of survival between different histologic grades.

**Histology grade**	**N**	**Mean (D)**	**Std error**	**Median (D)**	**Lower 95%**	**Upper 95%**	***p***
Low	12	1,161	270.5	1,112	336	1,818	
High (MI) >2	20	196.1	37.9	131^*^	55	279	<0.0001
Combined	32	558	131.6	285	130	495	

*Survival was significantly lower in the cases with a high mitotic index*.

* *Significant, p < 0.05*.

**Table 6:** Comparison of survival between different histologic types.

**Histology type**	**N**	**Mean (D)**	**Std error**	**Median (D)**	**Lower 95%**	**Upper 95%**	***p***
Epithelioid	15	790	240.5	356	131	1,141	
Spindle	4	528	186.6	369	290	1,083	
Mixed	12	313	148.3	94	41	495	0.035
Balloon cell	1	130	0	130	0	0	
Combined	32	558	131.6	285	130	495	

*The initial deceased survival detected in the mixed group was lost when using Bonferroni correction (p < 0.008) for multiple comparisons. MI, Mitotic index*.

## Discussion

We describe responses, overall survival and toxicity in a large group of dogs with a single tumor type, where there still is an unmet need regarding treatment modalities. Reported median survival time for dogs with stage II melanoma treated with curative intent surgery alone is less than five months, and in dogs with grade III or IV it is less than two to three months (1, 3, 39). In the current study, median survival for dogs in all stages was 285 days, whilst for dogs in stage II it was 336 days, and for stages III–IV was 128 days. Although this was not a randomized study aiming at proving efficacy, these survival times compare favorably to the previously reported figures. This suggests that AdCD40L immunotherapy has potential value in therapy of canine malignant melanoma CMM, either as adjuvant in a multimodal treatment setting, or for use as a primary therapy form when alternatives are not feasible. Finally, surgeries performed in this study were cytoreductive. In contrast to radical surgery, partial removal of the tumor leads to rapid recurrence locally, with short subsequent survival (39). The addition of AdCD40L immunotherapy as a consolidating therapy allowed survival comparable to situations when radical surgery is possible. Importantly, in contrast to radical surgery, immunotherapy plus cytoreductive surgery allowed high quality of life and functioning, with only mild and transient adverse reactions. Hence, the results encourage further investigation of the potential of adenoviral gene therapy in dogs with malignant melanoma.

Many different immunotherapy studies in canine malignant melanoma have been published (40). As many different techniques and case selection are involved, comparisons are hard to conduct. Different methodologies for gene transfer has been tested, such as CSPG4 (41), xenogenic Tyrosinase (42) and hgp100 (43). Collectively, macroscopic responses to immunotherapy are modest, but toxicity are tolerable and usually overall survival is reported to be favorable when combining with other therapies, preferentially surgery. To our knowledge, our studies are still the only one reporting responses in naturally occurring malignant melanomas in dogs using oncolytic viral therapy, although the technique is considering a promising modality also in dogs and many *in vitro* studies have been conducted (44, 45). The long-follow up time in this study is a strength and also rather unique, but comparable with at least one gene therapy clinical trial with a similar long-term follow-up published in the field (46). Oncolytic viral treatment in other canine malignancies are also reported (47).

A single pathologist (T.S.) reviewed the majority, but not all of the tumors. Since some of the tumors could not be re-evaluated, interpretation was done using the original histology reports. Currently, there is no consensus grading of canine malignant melanoma. Ramos-Vara and colleagues made the most promising attempt in 2000, using 338 oral malignant melanoma cases (38). Here several prognostic features were reported, and a score was described. Both in this study and as previously reported, proliferation stands out as a fairly robust prognostic indicator, where a mitotic index (MI) >2 is reported to shorten overall survival in many different types of malignant melanomas regardless of anatomic location (3, 38, 48). We therefore grouped the tumors into low and high grade if proliferation was reported to be MI >2. With this rather simple categorization, grade was prognostic with a median overall survival (OS) of 1,112 days with low-grade and only 131 days with high-grade tumors. One tumor was only diagnosed by cytology, but as the tumor was examined by a board-certified veterinary clinical pathologist and had so many cytological features of a high-grade malignant melanoma (and many visible mitotic figures), it was classified as an epithelioid high-grade malignant melanoma.

At least one clear case of abscopal effects of distant reaction in a CNS metastatic lesion was registered in the study. In future prospective studies with stronger funding allowing e.g. more frequent use of advanced digital imaging, the true occurrence of abscopal effect can be verified in dogs with melanoma treated with AdCD40L therapy. Comparing the OS data in this study with historic controls, especially as many dogs received no other treatment than immunotherapy for macroscopic malignant melanoma, the likelihood of abscopal effects in more dogs is, however, very high.

In human oncology several clinical trials have been conducted with AdCD40L therapy, prior to radical surgery (22, 49). (22, 49). Here, reference to the current dog study is of comparative value, as many of the dogs did not receive any surgery at all and the vast majority of those did not undergo radical surgery. These two factors have been reported to significantly worsen prognosis in dogs (39). A clinical trial with 15 treatment-refractory patients with malignant melanoma received intratumoral injections of AdCD40L (28). Nine of the patients also received low-dose cyclophosphamide conditioning before the first and fourth AdCD40L injection. As in dogs with malignant melanoma, the side effects were few with mild transient reactions. No macroscopic objective responses were recorded by MRI, but local and distant responses were seen on FDG-PET. Survival at six months appeared improved when cyclophosphamide was added to AdCD40L. The patients with the best survival developed the highest levels of activated T cells and experienced a pronounced decrease of intratumoral IL8. The results are encouraging for proceeding with adenoviral and CD40 directed treatment modalities.

No dogs were treated with immune interfering medication before AdCD40L injections. This enables a clean description of toxicity, but also the true efficacy of injections alone. Based on current understanding within immuno-oncology, the initial suppression of e.g. Tregs will potentiate the following immune therapy given. In one study, Mitchell and colleagues reported that the veterinary-registered TKI toceranib (Palladia, Zoetis, USA) had similar reduction potential of Tregs as classic low-dose cyclophosphamide (50). Toceranib is a multi-kinase inhibitor, closely related to sunitinib (51). The use of toceranib, as a conditioner in association with immune therapy in cancer patients, would also promote an antagonistic action toward VEGF, as well as add to the collective action to tilt the inflammatory environment in the tumor toward a less angiogenic environment with fewer immunosuppressive lymphocytes and more Th1 cells and M1 macrophages (1, 3, 38, 52–56).

The parameters found to be significant in association with OS were: Breed, Age, Stage, Grade, and Surgery together with immunotherapy.

Stage being a significant parameter complies with earlier reports (1–3). Considering that stage is a widely used prognostic tool it was expected that a correlation between stage and OS would be observed. This supports the validity of the WHO staging system as a prognostic tool. An expanded, revised staging system has been suggested, with more prognostic factors included (39). Herein the need to compensate for body weight is suggested, as a primary tumor with a diameter of 4 cm in a small-sized dog of 5 kg should be considered worse than the same tumor size in a dog weighing 40 kg. In our study we found a trend toward survival benefit for dogs weighing >20 kg (median OS of 495 vs. 225 days), although this was not statistically significant. If the study had been larger, the possibility of this finding being significant would likely increase. On the other hand, it is reported that increased bodyweight is negatively correlated with longevity in dogs (57).

Breed has also been shown in one previous study to be associated with OS (58). The most common breed in this study was the Golden Retriever, making up 6 of 32 patients, supporting the suggestion that this breed is particularly predisposed to CMM (38), although the patient group cannot be considered representative of the population as a whole because of the small sample size. No conclusions can be drawn in regard to specific breeds' positive or negative correlations with OS, although an unpowered significant difference was observed due to the fact that there were in most cases only one or two patients representing each breed.

Age was correlated to OS as dogs below 9y lived longer (*p* = 0.0119; Bonferroni correction (*p* < 0.012), when the dogs were categorized into three equal sized groups; <9, 9–11 and >11 years. Younger dogs have fewer co-morbidities and owner's may be more prone to invest in advanced treatments for younger animals. Still the significance is borderline and not interpreted as a major finding in this study.

Surgery showed a positive correlation with OS. This result may, however, be biased, for a number of reasons. Unfeasibility of radical surgery was one of the entry criteria for the clinical trial, emphasizing the exceptionally long survival time observed in the study. Whether or not a dog underwent surgery as part of treatment at a later stage may also have been influenced by a number of factors including the owners' personal feelings, age of the dog, previous treatment history, financial situation, etc. However, our results suggest that cytoreductive surgery may be useful in combination with immunotherapy for achieving the best results. The underlying mechanism may relate to the immunosuppressive nature of large tumors, partly conducted through tumor exosomes and generation of a pre-metastatic niche (59). Physically removing some of the immunosuppressive mass may facilitate the long-term effects of immunotherapy.

Tumors were staged according to the WHO staging scheme (36). The WHO staging, with a few exceptions, is divided into localization-specific tumor protocols. This study included tumors originating from cutaneous, oral, nasal, conjunctival and ungual tissue. However, due to a lack of specific protocol for conjunctival and ungual tumors according to the WHO staging scheme, these tumors were staged according to the criteria for dermal or epidermal tumors.

Spontaneous tumors in dogs have been suggested as good comparative models to human cancers. They occur spontaneously, in immunocompetent animals, often have similar or identical histologic appearance, share metastatic preferences and pattern, respond to the same type of treatment protocols (surgery, chemotherapy, radiotherapy and immunotherapy) and share the same environment as their owners (60–63). The pet dog has also been suggested as a good model to inform drug development and cancer treatment trials in humans (64). The comparative aspects of malignant melanoma in dogs have been extensively reviewed, showing both molecular biological similarities and thanks to a spontaneous developing tumor in an immune competent animal, ideal for investigating new treatment modalities, especially immunotherapy (10, 11, 14, 65). Finally, the rapidly increasing resolution of the dog genome sequence makes canine comparative oncology studies easier to perform with high accuracy (66).

The findings in this study, with encouraging survival data, low toxicity and the achievement of good quality of life during and post treatment in such a complicated tumor in dogs, supports the usefulness of the canine melanoma model for developing and informing parallel clinical studies in humans with malignant melanoma.

## Data Availability Statement

The raw data supporting the conclusions of this article will be made available by the authors, without undue reservation.

## Ethics Statement

The animal study was reviewed and approved by Swedish Animal Ethical Committee and the Swedish Animal Welfare Agency (C228/6). Written informed consent was obtained from the owners for the participation of their animals in this study.

## Author Contributions

SS, MD, and HR treated and monitored the patients at the animal hospital, AH, AL, OK, TT, SS, and HR contributed to conception and design of the study. SS, HR, and AH organized the database and performed the statistical analysis. AS, EE, AL, and TT performed the *in vitro* studies and lab work. TS performed the pathology examinations. SS wrote the first draft of the manuscript. HR, AL, and AH wrote sections of the manuscript. All authors contributed to manuscript revision, read, and approved the submitted version.

## Conflict of Interest

AH is a shareholder in Targovax ASA. AH is an employee and shareholder in TILT Biotherapeutics Ltd. AL is the CEO of Lokon Pharma AB, a shareholder of Lynxalo AB, a board member of Lokon Pharma AB, Vivolux AB, Repos Pharma AB, Tanea Medical AB, Bioimics AB, Lynxalo AB and an alternate board member of Nexttobe AB, Almoalo AB, Promegranate Veterinary AB and Aros Biotech AB. Further, AL has a royalty agreement with Alligator Bioscience AB. The remaining authors declare that the research was conducted in the absence of any commercial or financial relationships that could be construed as a potential conflict of interest.

## Publisher's Note

All claims expressed in this article are solely those of the authors and do not necessarily represent those of their affiliated organizations, or those of the publisher, the editors and the reviewers. Any product that may be evaluated in this article, or claim that may be made by its manufacturer, is not guaranteed or endorsed by the publisher.
